# The role of complex interactions between the intestinal flora and host in regulating intestinal homeostasis and inflammatory bowel disease

**DOI:** 10.3389/fmicb.2023.1188455

**Published:** 2023-06-14

**Authors:** Siyu Li, Kan Xu, Yi Cheng, Lu Chen, Ailin Yi, Zhifeng Xiao, Xuefei Zhao, Minjia Chen, Yuting Tian, Wei Meng, Zongyuan Tang, Shuhong Zhou, Guangcong Ruan, Yanling Wei

**Affiliations:** ^1^Department of Gastroenterology, Chongqing Key Laboratory of Digestive Malignancies, Daping Hospital, Army Medical University (Third Military Medical University), Chongqing, China; ^2^Basic Medicine College of Army Medical University, Army Medical University, Chongqing, China; ^3^Department of Laboratory Animal Center, Daping Hospital, Army Medical University (Third Military Medical University), Chongqing, China

**Keywords:** intestinal flora, intestinal homeostasis, inflammatory bowel disease, short chain fatty acids (SCFAs), functional foods, gut metabolome, IBD therapies

## Abstract

Pharmacological treatment of inflammatory bowel disease (IBD) is inefficient and difficult to discontinue appropriately, and enterobacterial interactions are expected to provide a new target for the treatment of IBD. We collected recent studies on the enterobacterial interactions among the host, enterobacteria, and their metabolite products and discuss potential therapeutic options. Intestinal flora interactions in IBD are affected in the reduced bacterial diversity, impact the immune system and are influenced by multiple factors such as host genetics and diet. Enterobacterial metabolites such as SCFAs, bile acids, and tryptophan also play important roles in enterobacterial interactions, especially in the progression of IBD. Therapeutically, a wide range of sources of probiotics and prebiotics exhibit potential therapeutic benefit in IBD through enterobacterial interactions, and some have gained wide recognition as adjuvant drugs. Different dietary patterns and foods, especially functional foods, are novel therapeutic modalities that distinguish pro-and prebiotics from traditional medications. Combined studies with food science may significantly improve the therapeutic experience of patients with IBD. In this review, we provide a brief overview of the role of enterobacteria and their metabolites in enterobacterial interactions, discuss the advantages and disadvantages of the potential therapeutic options derived from such metabolites, and postulate directions for further research.

## 1. Introduction

IBD is a group of autoimmune-related intestinal inflammatory diseases. With worldwide development, the incidence of IBD is increasing worldwide and represents an increasingly heavy burden for society (Kaplan and Windsor, 2021). Current clinical treatment for IBD is inefficient, and patients with good early efficacy must face the dilemma of drug withdrawal (Chapman et al., 2020). More potential therapies are being considered, including antibiotics and biological inhibitors.

The composition and basic functions of enterobacteria have been researched for decades and enterobacteria play an important role in maintaining host homeostasis. Unlike the previously held gut-dominated model of action, the new model of entero-bacterial interactions highlights the important role of both enterobacteria and the intestinal environment and is gaining increasing interest. The intestinal flora is thought to play a key role in triggering IBD, and dysbiosis is strongly associated with IBD. Enterobacterial metabolites play an equally important role in disease incidence, and their disruption is thought to contribute to the pathogenesis of IBD through a variety of immune and pro-inflammatory pathways (Lavelle and Sokol, 2020). In addition, genetic defects associated with IBD and environmental factors can induce the accumulation and invasion of pathogenic bacteria in the intestinal tissues, thus further promoting dysbiosis and inflammation (Lavelle and Sokol, 2020). However, the mechanisms associated with enterobacterial interactions have not been elucidated, and the factors influencing enterobacterial interactions have not been specifically summarized.

The causal relationship between intestinal disorders such as IBD and intestinal flora has been of particular interest outside of specific animal models. However, the relationships remain elusive, and therapeutic tools acting on enterobacterial interactions are limited. Many reports suggest that the composition and balance of the microbiota and its metabolites are altered in IBD (Manichanh et al., 2006; Vich Vila et al., 2018; Lo Presti et al., 2019; Ryvchin et al., 2021; Čipčić Paljetak et al., 2022). In addition, the effect of antibiotic administration in the gut of IBD patients on disease progression is varies widely depending on the population and the type of antibiotic used, suggesting that alterations in the intestinal flora may be associated with disease progression or remission (Hviid et al., 2011; Ledder and Turner, 2018; Breton et al., 2019; Nguyen et al., 2020). Some intestinal flora metabolites are characteristically reduced in the stool of Crohn’s disease (CD) and ulcerative colitis (UC) patients (Marchesi et al., 2007). These studies suggest a possible role for antibiotics in improving the gut microbiota and metabolites in treating IBD but do not suggest a specific viable treatment. For example, the effect of antibiotics on IBD is often heterogeneous, and it is controversial whether the efficacy of different antibiotics in different patients outweighs the side effects of antibiotic use; this controversy is more evident in patients with UC (Ianiro et al., 2016; Ledder and Turner, 2018).

In recent years, various proposals for altering the gut microbiota have been tested to treat IBD, including fecal transplantation to replace the flora and the addition of probiotics with anti-inflammatory properties (Sood et al., 2009; Damman et al., 2012; Jakubczyk et al., 2020). Some of these ideas are well established and have entered clinical use; some probiotics are effective in a mouse model induced by dextran sodium sulfate (DSS) and in patients with mild to moderate UC (Sood et al., 2009; Wang et al., 2018). In this review, we will summarize the roles of enterobacteria and their metabolites in IBD and provide a summary of IBD therapies targeting enterobacteria in recent years.

## 2. Microbial dysbiosis in IBD

The composition of each microbial community varies significantly between individuals and is highly heterogeneous. Even between identical twins, only 40% of bacteria strains may be shared (Turnbaugh et al., 2010). The microbial community undergoes constant adaptations to the intestinal environment and function, such as the availability of lactic acid bacteria in newborns following delivery to help digest lactose and other substrates that infants cannot digest (Zivkovic et al., 2011). This adaptation is also reflected in the altered microflora of IBD (Ni et al., 2017).

### 2.1. Changes in intestinal flora in patients with IBD

Many studies have reported significant reductions in enterobacterial biodiversity in intestinal tissue samples from patients with IBD, as evidenced by reductions in the total number of species in the community. Table 1 presents the gut microorganisms that have been reported to play a significant role in enterobacterial interactions in IBD. Sepehri et al. reported that the gut flora in different parts of the intestine of IBD patients exhibited significantly reduced biodiversity compared to healthy individuals; furthermore, these differences were significantly associated with the degree of disease activity (Sepehri et al., 2007). Clostridium subsets in the phylum Firmicutes, such as cluster IV and subcluster XIVa, as well as Lachnospiraceae and Bacteroides were reduced in IBD, whereas parthenogenetic anaerobes, actinomycetes, Aspergillus, and phylum Proteobacteria increased (Frank et al., 2007; Macfarlane et al., 2009; Nishino et al., 2018; Lloyd-Price et al., 2019). The abundance of each group again differed in UC compared to CD patients. For example, the abundance of the phyla Firmicutes and Bacteroidetes increased (Macfarlane et al., 2009; Forbes et al., 2016). Within these phyla, species considered to have damaging effects on the intestinal mucosa, such as Escherichia, the genus Ruminococcus (*R. gnavus*) and Fusobacterium, became dominant, replacing *Clostridium difficile*, which protects the intestinal mucosa (Sepehri et al., 2007). Over recent decades, the microflora alterations in IBD patients have been well documented, including changes in phylum Firmicutes and butyric acid-producing bacteria. However, whether most species change in abundance between IBD and healthy populations and between UC and CD patients is widely debated (Macfarlane et al., 2009; Forbes et al., 2016). Previously observed alterations may also be related to selecting samples from different regions and populations. DGGE mapping of the 16S gene suggests that although environmental factors are essential, human genetics is the primary driver of enterobacterial alterations (Zoetendal et al., 2001).

**Table 1:** Reported roles of gut microbes in gut-bacteria interactions.

Researcher	Risk factor	Species affected	Changes in IBD	Model	Type of sample	Mechanism	References
Matthew R. Kudelka	Molecular chaperone *Cosmc* delete in IECs	Bacteroides, Helicobacter	Decreased	Human, mice	Distal colon	Lipocalin-2 increased, crypt abscesses and inflammatory infiltrate limited in distal colon.	Kudelka et al. (2016)
Suzanne Devkota	IL-10(−/−)	*B. wadsworthia*	Increased	Mice	Distal colon	Release H_2_S to break barriers; Associated with T_H_1 immunity	Devkota et al. (2012)
Liu H.	Atg16L1(T300A/T300A)	Akkermansia	Decreased	Mice	Distal colon	Mucin-degrading bacterium; Protect intestinal epithelium and mucosal function	Liu et al. (2021)
		Tyzzerella, Mucispirillum, Ruminococcaceae	Increased			Opportunistic pathogens	
R. Caruso	NOD2 and *CYBB* deleted in monocytes	*Mucispirillum schaedleri*	Increased	Mice	Distal colon	Positive associated with fecal levels of Lcn-2, colon shorten intestinal inflammation	Caruso et al. (2019)
Yasunori Ogura	NOD2 deleted in monocytes	*Salmonella typhimurium*, *Shigella flexneri 1A, Klebsiella pneumoniae, Campylobacter jejuni*	Decreased	Human	Human embryonic kidney (HEK) 293 T cells	Release LPS to activate NOD2 expressing NF-κB	Ogura et al. (2001)
Eran Elinav	Deficiency of NLRP6 in mouse colonic epithelial cells	phyla Bacteroidetes (Prevotellaceae)	Increased	Mice	Distal colon	Intestinal hyperplasia, inflammatory cell recruitment, leading to exacerbation of DSS colitis via induction of CCL5	Elinav et al. (2011)
Monika Schaubeck	TNF (deltaARE)	*Escherichia coli* LF82	Increased	Mice	Distal colon	Triggered loss of lysozyme and cryptdin-2 expression, inducing inflammation	Schaubeck et al. (2016)
Daniel N. Frank		Bacteroidetes, Lachnospiraceae subgroup of Firmicutes	Decreased	Human	Colon, small intestine	Enhance epithelial barrier integrity and modulate the GI immune response	Frank et al. (2007)
		Proteobacteria, Bacillus subgroup of Firmicutes	Increased			(Unannounced)	

### 2.2. Role of the intestinal flora in IBD through the immune response

The causal relationship between altered intestinal flora diversity and the pathogenesis of IBD has not been elucidated. Most studies have focused on the interaction between microorganisms and IBD through immune responses, with a healthy gut regulating intestinal ecology and building intestinal mucosal barriers through the secretion of antimicrobial peptides, IgA, and mucins (Grigg and Sonnenberg, 2017). It is demonstrated that IL5, IL13, IL17, and IL23 expression is increased and IL-33 expression is reduced in IBD patients (Kobori et al., 2010; Rosen et al., 2017). Among these cytokines, IL-23 is considered to have functional role. Stimulation of colonic leukocytes with IL-23 induced the production of IL-17, which has an association with bacteria-driven innate colitis. One additional evidence is that, it is known that innate immune colitis in Rag−/− mice following infection with *Helicobacter hepaticus* is IL-23 dependent, and IL-23R expression is controlled by transcription factor ROR-gammat, while Rag−/−Rorc−/− mice failed to develop innate colitis, suggesting that IL-23 has an positive effect on improving IBD (Hue et al., 2006; Buonocore et al., 2010; Ermann et al., 2014; Hall et al., 2017). Elinav et al. demonstrated that NLRP6 deficiency in mouse colonic epithelial cells resulted in reduced IL-18 levels and altered fecal microbiota characterized by expanded representation of the bacterial phyla Bacteroidetes (Prevotellaceae) and TM7, which in turn induces IBD pathogenesis (Elinav et al., 2011). Intestinal microbiota metabolites taurine, histamine, and spermine also shape the host-microbiome interface by co-modulating NLRP6 inflammasome signaling, epithelial IL-18 secretion, and downstream anti-microbial peptide (AMP) profiles, suggesting the dual role of intestinal flora and IBD (Levy et al., 2015).

Nevertheless, downstream mechanisms of a specific inflammatory factor is often unclear for it is influenced by multiple factors; whether alterations in that factor are influenced more by the disease itself or by microorganisms has yet to be explored in depth. For example, IL-33 induces ST2 constitutively expressing in immune cells, allowing IL-33/ST2 axis to act as a bridge between immune system orchestration and tissue injury. Kinchen et al. found that the pro-inflammatory factor IL-33, one of the markers of the S4 subpopulation of intestinal mesenchymal cells, which activates in dysregulation of niche in IBD, exhibits increased expression in the intestinal mucosal tissue of both human and mouse patients with IBD, and induced factors that impaired epithelial proliferation and maturation and contributed to oxidative stress and disease severity *in vivo* (Kinchen et al., 2018). IL-33 also collaborates with TLRs in promoting pro-inflammatory cytokine responses through the disruption of tolerogenic responses against intestinal bacteria. However, Malik et al. reported that IL-33 levels are reduced in IBD patients. In contrast, increased levels of mucolytic and colitogenic bacteria (e.g., *Akkermansia*) in IL-33-deficient mice were reported to contribute to the development of colitis by promoting increased levels of IL-1α. IL-33 was therefore suggested to act by inhibiting the development of IBD (Malik et al., 2016). This relationship illustrates the limitations of studies on particular inflammatory factor signaling pathways, and suggests the need to evaluation of “when” and “how” the IL-33/ST2 signaling when exploring novel IL-33-targeting biological agents in the therapeutic armamentarium against IBD in the future (Aggeletopoulou et al., 2022).

### 2.3. Factors affecting the interaction between intestinal flora and IBD

Most studies have focused on the microbiota as one of multiple IBD triggers rather than studying it as a single influencing factor. Several factors influence the interaction between microorganisms and IBD, including human genetic and nutritional perspectives are generally accepted.

#### 2.3.1. Genetic polymorphisms

Over 250 bacterial species are susceptible to genetic changes in IBD, including Atg16L1, NOD2, NLRP6, and others (Agrawal et al., 2022). Compared to WT mice, cup cells in Atg16L1^T300A/T300A^ mice exhibited reduced mucin secretion due to defective autophagy, whereas the mucin-degrading bacterium *Akkermansia*, which renews the mucosal layer and protects the intestinal barrier, was significantly less abundant in Atg16L1^T300A/T300A^ mice (Cadwell et al., 2008; Liu et al., 2021). These mice also exhibited a significantly increased abundance of *Ruminococcaceae* associated with IBD (Liu et al., 2021). Combined deletion of the NOD2 gene and phagocytic NADPH oxidase (CYBB) gene leads to an increased abundance of *Mucispirillum schaedleri*, which in turn causes TNFα-dependent early spontaneous TH1 enteritis (Ogura et al., 2001; Caruso et al., 2019). One case–control analysis reported that healthy individuals with high genetic risk defined as 11 functional genetic variants including NOD2 and ATG16L1 that was regarded as genes that are directly involved in the bacterial handling in the gut, is significantly associated with a decrease in the genus *Roseburia* in healthy controls, whose metabolisms has been shown to induce Treg cells, preventing or ameliorating intestinal inflammation. The characteristic alteration of NLRP6 deficiency in mouse colonic epithelial cells is that exposure to DSS worsens colitis, and the resultant increased abundance of phylum Bacteroidetes (Prevotellaceae) can produce DSS-induced colitis by inducing CCL5 in neonatal or adult wild-type mice (Elinav et al., 2011). The development of Crohn’s disease-like ileitis is microbially dependent in TNF^deltaARE^ mice, and transfer of the microbiota from CD mice with dysregulated intestinal ecology to genetically susceptible sterile recipient mice induces Crohn’s disease-like inflammation, demonstrating a triggering role for the microbiota in the pathogenesis of genetically susceptible IBD patients (Schaubeck et al., 2016). cGAS knockout mice exhibit reduced levels of Desulfovibrio spp., Enterococcus spp. and *Escherichia coli* compared with DSS-induced IBD model mice; furthermore, cGAS knockout mice are less susceptible to DSS-induced colitis, suggesting a relationship between the cGAS gene in altering intestinal ecology and IBD pathogenesis (Wang Z. et al., 2021). The common feature of these genes is that they can alter intestinal ecology and cause or ameliorate the onset of colitis through the secretion of cytokines or alterations in the intestinal environment; this action is reciprocal, demonstrating the important role of inflammatory factors in entero-bacterial interactions. Notably, these mechanistic studies have been performed only in animal models. There are more genes, even immune-associated genes, may act on the intestinal flora through other pathways. One recent clinical research identified the involvement of IBD-related genes in the regulation of the intestinal microbiota (Hu et al., 2021), indicating a significant difference in the phenotype of the immune-related gene CABIN1 compared to healthy controls, which is associated with an increase in the D-glucaric acid degradation pathway. Interestingly, enterobacteria such as *E. coli*, a potentially pathogenic bacterium known to be enriched in dysregulated conditions, can use this sugar as a carbon source for growth (Gulick et al., 2001).

Gene-based burden test of microbial quantitative trait loci showed that host genes LEKR1, CYP2D6, GPR151, and TPTE2 in IBD patients could regulate bacterial metabolic pathways, such as inhibition of the hexanol-degrading bacterial superpathway, glucose 6-phosphate glycolytic pathway, and inhibit bacterial biosynthesis of vitamin K, among other bacterial biosynthesis levels (Hu et al., 2021).

COSMC controls the extension of O-glycans beyond a single GalNAc, which has a direct interface with the gut microbiota. To test the hypothesis that Cosmc spatially regulates the gut microbiome, Kudelka et al. deleted COSMC in the mouse intestine, which led to loss of microbial diversity and emergence of a pathobiont in the mucosa of the distal colon, but not in the overlying lumen or in the mucosa of the small intestine, determining that COSMC and the downstream O-glycans spatially regulate the gut microbiome (Kudelka et al., 2020).

Comparing the methylation of different genes across microbiota clusters, researchers observed 33 and 19 significantly hyper-methylated or hypo-methylated sites in cluster, respectively, including hyper-methylated signals in the gene body of NOTCH4, and hypo-methylation in CCDC88B and TAP2 (Ryan et al., 2020). Mechanism of interaction between methylation signals and microbiota alter is not clear as far.

#### 2.3.2. Diet

Diet directly influences the development of IBD, e.g., dextran sodium sulfate (DSS; Wirtz et al., 2017). Similarly, a hypo-methyl diet methyl-deficient diet (MDD) has also been shown to exacerbate DSS-induced IBD (Melhem et al., 2016). The progression of IBD can be influenced by different dietary structures and a variety of nutrients, as described later. A meta-analysis of 12 randomized controlled trials (*n* = 611) and 4 observational studies (*n* = 359) noted that vitamin D supplementation in patients with IBD was effective in correcting their vitamin D levels, reducing C-reactive protein levels, and decreasing intestinal inflammatory activity, suggesting a role for vitamin D in the pathogenesis of IBD (Guzman-Prado et al., 2020). Another mechanism of action is that the alpha diversity and abundance of intestinal flora are regulated by diet, particularly in the case of certain strains of bacteria that are closely associated with the development of IBD, which may also be one of the mechanisms of action in IBD. Researchers in food science typically use a single nutrient as a single influencing factor; for example, vitamin A supplementation was found to antagonize the intestinal damaging effects of LPS and protect the intestinal mucosal layer, whereas Akkermansia renews the protective layer of the intestinal mucosa, and its abundance decreases in the intestine of vitamin A-deficient individuals. Investigations into the interaction between a food component as a prebiotic or enterobacterial metabolite and the intestine have also been undertaken, as described later (Vitamin A inhibits the action of LPS on the intestinal epithelial barrier function and tight junction proteins; the role of genotype and diet in shaping gut microbiome in a genetic vitamin A deficient mouse model). Different lines of research focus on producing different guidance for different populations, such as dietitians or gastroenterologists. However, regardless of the line of research, the cause-and-effect relationships and mechanisms underlying the effects of diet on the gut and the effects of diet on the intestinal flora are often difficult to describe and require additional basic experimental evidence.

#### 2.3.3. Other

Experimental stress in animals increases intestinal mucosal permeability and also alters bacterial-host interactions (Mawdsley and Rampton, 2005). One hour sessions of water avoidance stress for 10 consecutive days increased the phagocytic uptake of killed *Escherichia coli* into follicle associated epithelium in mice (Velin et al., 2004). And as one of the mechanisms that genes take effect on interaction between intestinal flora and IBD, the increase in intestinal permeability associated with the stress response might also allow the gut microbiota to interact with the nervous system; which is so-called microbiota-brain-gut axis (Gracie et al., 2019). Moreover, exosomes derived from infected immune cells also induce a specific anti-microbial immune response through displaying antigen (De Toro et al., 2015). Actually, Exosomal Proteins can regulate intestinal barrier function and intestinal flora, respectively, in different situations. For example DC-derived exosomal heat shock protein 73 (HSP73) and IEC-derived exosomal HSP72 have already been identified that can utilize receptors for both Gram-positive (TLR2) and negative bacteria (TLR4) to stimulate the proinflammatory signal in a CD14-dependent fashion (Asea et al., 2002). On the other hand, ANXA1 is one potent endogenous pro-resolving mediator that released from IECs into the extracellular space, while ANXA1 mimetic peptide, Ac2-26, accelerates the recovery of epithelial barrier function (Leoni et al., 2015).

## 3. Gut metabolome in IBD

The intestinal metabolome of IBD patients is disturbed, affecting immunity, inflammation and gene expression. These changes manifest as metabolic dysregulation of short-chain fatty acids, bile acids and tryptophan. The following describes the changes in several major metabolites in IBD and their mechanisms (Table 2).

**Table 2:** Primary alterations in gut microbial metabolites and their underlying causal mechanism in IBD.

Subjects		Changes in IBD	Mechanism	References
SCFAs	Acetate	↓	Activation of GPR43	Marchesi et al. (2007) and Masui et al. (2013)
	Propionate	↓	Activation of CD4 T cells and Tregs and IL-10	Smith et al. (2013) and Xia et al. (2017)
			Activation of ZO-1, claudin-1, claudin-8, occludin	
	Butyrate	↓	Production of IL-22 by CD4 T cells and ILCs and through GPR41 and HDAC inhibition and activation of Stat3 and mTOR	Marchesi et al. (2007), Chang et al. (2014), Chen G. X. et al. (2018), Yang et al. (2020), and Hansen et al. (2021)
			Activation of ZO-1, occluding, claudin-1, JAM-3	
			Inhibition of NO, IL-6, IL-12p40 and HDAC in macrophage	
Bile acid	PBA	↑	Inversely correlated with SCFAs-producing bacteria	Fiorucci et al. (2021) and Li N. et al. (2021)
	CA	↑	Activation of FXR to inhibit NF-κB and inflammatory cytokines and produce antimicrobial peptides	Ding et al. (2015) and Li N. et al. (2021)
	CDCA	↑	Activation of FXR to inhibit NF-κB and inflammatory cytokines and produce antimicrobial peptides	Ding et al. (2015) and Li N. et al. (2021)
	SBA	↓	Positively correlated with SCFAs-producing bacteria	Ding et al. (2015) and Li N. et al. (2021)
	DCA	↓	Activation of Tregs through induction of Foxp3 by inhibiting DC	Ding et al. (2015), Lavelle and Sokol (2020), Sorrentino et al. (2020), Li N. et al. (2021), and Cai et al. (2022)
			Activation of TGR5 and regeneration of intestinal stem cells	
			Activation of FXR to inhibit NF-κB and inflammatory cytokines and produce antimicrobial peptides	
			Inhibition of IL-8 induced by IL-1β	
	LCA	↓	Inhibition differentiation of TH17 through RORγt and mitoROS	Ding et al. (2015), Hang et al. (2019), Fiorucci et al. (2021), Li N. et al. (2021), and Cai et al. (2022)
			Activation of Tregs through Foxp3 Inhibition of NF-κB and inflammatory cytokines	
			Activation of TGR5 and regeneration of intestinal stem cells	
			Activation of FXR to inhibit NF-κB and inflammatory cytokines and produce antimicrobial peptides	
Tryptophan	Tryptophan	↓	Inhibition of IL-8 induced by IL-1β	Lamas et al. (2016) and Lavelle and Sokol (2020)
	KYN	↑	Activation of IL-6 via AHR in IEC and inhibition in macrophages	Lamas et al. (2016) and Wang D. et al. (2021)
			Activation of IDO1 with IL-6	
			Phosphorylation of JAK2 and STAT3	
	KYNA	↑	Activation of AHR and AHR-IL-22 axis	Elizei et al. (2017), Wang D. et al. (2021), and Michaudel et al. (2022)
			Activation of mitochondrial metabolism in IEC and glycolysis of T cells	
			Induction of T-cell differentiation to TH17	
			Inhibition of IL-23p19 in DC via activation of GPR35 and reduction of cAMP	
			Inhibition of IL-17 and reduction of TH17	
			Activation of IL-6 via AHR in IEC and inhibition of macrophages	
			Activation of IDO1 with IL-6	
			Phosphorylation of JAK2 and STAT3	
	Quinolinic acid	↑		Nikolaus et al. (2017)
	5-HT	↓ ↑	Induction of macrophage polarization (upregulation of M2 and downregulation of M1)	de las Casas-Engel et al. (2013), Sikander et al. (2015), Spohn et al. (2016), Banskota et al. (2017), and Coates et al. (2017)
			Induction of epithelial proliferation via 5-HTR4	
			Activation of NF-κB, TLR and IL-8 via activation of NOX2	
	Indole	↓	Activation of GJE1, GJB3, GJB4, GJA8 and muc1	Bansal et al. (2010) and Alexeev et al. (2018)
			Inhibition of IL-8and NF-κB	
	IPA	↓	Activation of IL-10R1 in IEC and stem cells	Venkatesh et al. (2014) and Alexeev et al. (2018)
			Activation of transcription of AHR	
			Inhibition of TNF-α and activation of junctional protein	
			Activation of PXR	
	IAld	↓	Induction of SOCS3 with IL-10 Activation of IL-10R1 in IEC via AHR	Alexeev et al. (2018)
	IAA	↓		Lamas et al. (2016)

SCFAs, short-chain fatty acids; PBA, primary bile acids; CA, cholic acid; CDCA, chenodeoxycholic acid; CBA, conjugated bile acids; SBA, secondary bile acids; DCA, deoxycholic acid; LCA, lithocholic acid; UDCA, ursodeoxycholic acid; KYN, kynurenine; KYNA, kynurenic acid; IPA, indole-3-propionic acid; IAld, indole-3-aldehyde; IAA, indole-3-acetic acid; FXR, organic solute transporter α/β. ^**^The potency of FXR activation is CDCA > LCA = DCA > CA (Yang et al., 2020).

### 3.1. SCFAs

Numerous studies have demonstrated the presence of reduced short-chain fatty acids (SCFAs) in the stool of patients with IBD. In a population-based analysis, acetic acid and butyric acid were found to be significantly lower in IBD patients than in normal subjects (Marchesi et al., 2007). SCFAs are a group of saturated fatty acids containing 2–5 carbon atoms, of which acetic acid (C2), propionic acid (C3), and butyric acid (C4) are the most abundant, accounting for approximately 90% to 95% (Ma et al., 2022). SCFAs are the main products of fermentation of dietary fiber by anaerobic bacteria colonizing the colon and cecum (Venegas et al., 2019). SCFAs are primarily produced by bacteria such as Acinetobacter, Bifidobacterium, and Bacteroides (Bpp.), Bifidobacterium (Dialister spp.), Coproestreeutacus, *C. comes*, and AnAerostipe spp. (Peng et al., 2022). After analyzing 127 UC and 87 healthy individuals, there was a significant reduction in butyric acid-producing bacteria *R. hominis* and *F. prausnitzii* in UC feces (Machiels et al., 2014). Butyrate also increases flora diversity in the treatment of DSS mice, resulting in a decrease in the abundance of fecal anaplasma and an increase in Firmicutes and is increasingly used in the treatment of IBD (Simeoli et al., 2017; Dou et al., 2020) SCFAs have been shown to improve the pathophysiological process of IBD by strengthening the intestinal barrier, suppressing the inflammatory response, modulating the immune response, and influencing intestinal microbes. SCFAs promote the production of the intestinal interepithelial tight junction proteins occludin, zonula occludens (ZO), and claudins, promote intestinal cell proliferation, and increase MUC3 and MUC5B expression, thereby strengthening the epithelial and mucus barriers and reducing intestinal permeability (Gaudier et al., 2004; Venegas et al., 2019; Ma et al., 2022). SCFAs signal primarily through G-protein-mediated signaling pathways, activating G protein receptors (GPCRs) on cell membranes, including GPR41, GPR43, and GPR109a. Activation regulates migration, growth, value-added, differentiation, apoptosis, and cytokine release of immune cells, and GPCRs are present in the membranes of intestinal cells and a variety of immune cells (Kim et al., 2013; Macia et al., 2015). An experiment demonstrates that SCFAs can promote IL-22 production by CD4+ T cells and ILCs through GPR41 and HDAC (histone deacetylase) and are regulated and mediated by HIF1α (Hypoxia Inducible Factor1α), AhR, stat3, and mTOR, which act as inhibitors of inflammation.

Another study reported that the addition of 150 mM acetic acid to drinking water ameliorated intestinal inflammation in DSS (Dextran Sulfate Sodium Salt) mice, resulting in an increase in colon length and a decrease in the disease activity index, where there was no significant therapeutic effect in GPR43 knockout mice, suggesting that acetic acid can regulate colitis through GPR43 (Masui et al., 2013). SCFA binding and subsequent GPR43-induced neutrophil chemotaxis and Treg proliferation. Acetic acid further, downregulates the inflammatory response and improve colonic inflammation in IBD patients (Smith et al., 2013; Furusawa et al., 2014). SCFAs also inhibit HDAC through GPCRs, promote histone acetylation, and regulate gene expression (Tan et al., 2014; Peng et al., 2022). Increased histone acetylation was observed in the inflamed mucosa of both CD patients and TNBS-induced (Trinitro-benzene-sulfonic acid) and DSS-induced mouse intestines, suggesting a possible association between histone acetylation and intestinal inflammation (Tsaprouni et al., 2011). SCFAs inhibited HDAC, reduced TNF expression in monocytes and neutrophils, inhibited the NF-κB pathway, and suppressed the expression of proinflammatory factors such as IL-2, IL-6, and IL-8. In Treg cells, SCFAs promoted FOXP3 expression and inhibited proteasome degradation, thereby suppressing the inflammatory response; in intestinal macrophages, SCFAs inhibited the production of the proinflammatory substances NO, IL-6, and IL-12p4064 (Ma et al., 2022). As previously described, disturbances in intestinal microecology are important factors in the pathogenesis of IBD, and there is growing evidence that studies on the mechanisms of action between SCFAs and intestinal microbes may provide the basis for human cohort studies.

SCFAs are diverse and regulate the pathogenesis of patients with IBD in various ways, including affecting the intestinal barrier, modulating inflammatory and immune responses, and regulating intestinal microecology. However, further mechanisms by which SCFAs affect IBD and the mechanisms of different species are not well understood. The close association of SCFAs with IBD provides a new means of treating IBD; however, current studies have mainly focused on butyrate, and there is a lack of data from studies of multiple SCFAs and safety date. Therefore, further research is urgently needed to elucidate the mechanism of SCFA actions and validate them as a therapeutic tool.

### 3.2. Bile acids

Cholesterol undergoes a series of enzymatic reactions in the liver and intestine to produce primary bile acids (PBAs) and secondary bile acids (SBAs), which are utilized by the body (Di Vincenzo et al., 2022). In patients with IBD, fecal PBA, bile acids (CA), goose deoxycholic acid (CDCA), and CBA are increased and SBA, deoxycholic acid (DCA), and stone bile acids (LCA) are decreased compared with normal subjects (Fiorucci et al., 2021; Li N. et al., 2021). The increase in PBA and decrease in SBA can be used as diagnostic markers for IBD. Yang et al. analyzed the fecal flora and metabolites of 32 UC patients and 23 normal subjects and found that the UC group had a lower fecal flora alpha diversity index as well as a lower SBA and significantly higher PBA than controls. Furthermore, the UC group exhibited a lower abundance of Clostridium IV and *Clostridium butyricum*, whereas Proteus and Ehrlichia spp. were significantly more abundant (Yang Z. H. et al., 2021).

There is an intrinsic bidirectional relationship between impaired bile acid absorption and IBD through dysregulated expression of ASBT and OSTα/β, which are important channels for the passage of bile acids from enterocytes into the blood. Alterations in the expression of these channels leads to reduced bile acid absorption (Macierzanka et al., 2019; Fitzpatrick and Jenabzadeh, 2020). mRNA expression of apical sodium-dependent bile acid transporter (ASBT) and organic solute transporter α/β is reportedly downregulated in patients with CD and UC, suggesting a relationship between reduced bile acid absorption and IBD (Jahnel et al., 2014). FXR is an important nuclear receptor for BAs and exhibits the highest binding affinity to hydrophobic Bas, with an affinity as follows: CDCA> LCA = DCA > CA (Ding et al., 2015). BAs bind to FXR on IECs and leads to the secretion of FGF19, which inhibits bile acid production. Levels of FGF19 in patients with Crohn’s disease is lower than in healthy controls (Fiorucci et al., 2021). The activation of FXR regulates the expression of ASBT, ileal bile acid binding protein (I-BABP) and OSTα/β, thus regulating the entire process of bile acids from the intestine to the liver (Tiratterra et al., 2018). In addition, FXR activation promotes the expression of iNO, ANG1, and CAR12 and inhibits proinflammatory factors such as *TNFα*, IL-1, IL-1β, IL-6, MCP-1, IL-17, and IFNγ. Furthermore, FXR activation inhibits inflammation, reduces damage to the intestinal barrier, and alleviates clinical signs of weight loss, rectal bleeding, shortened colon and reduced intestinal permeability in DSS mice (Inagaki et al., 2006; Gadaleta et al., 2011; Ding et al., 2015). The extracted BA mixture promotes the regeneration of the intestinal epithelium by stimulating TGR5 in intestinal stem cells, thus activating SRC and YAP and their target genes to promote the regeneration of intestinal epithelial injury (Sorrentino et al., 2020). Lithocholic acid (LCA) and its metabolites inhibit the differentiation of TH17 cells and promote the differentiation of Treg cells through RORγt and mito ROS, which directly regulate the host immune response. LCA and metabolites also inhibit the production of NF-κB and proinflammatory factors to suppress inflammation (Hang et al., 2019). The effect of disturbances of the intestinal flora on bile acid metabolism in the IBD population is complex, and butyric acid-producing bacteria in feces are reported to be positively correlated with SBA and negatively correlated with PBA (Li N. et al., 2021). BSH is primarily derived from Firmicutes, followed by *Bacteroides mimicans* and Actinobacteria. However, the Firmicutes population was significantly lower in CD patients, and no change was observed in Firmicutes and Actinobacteria; Actinobacteria populations were significantly higher in UC patients, and no change was observed in Firmicutes and Actinobacteria (Ogilvie and Jones, 2012). CA increased the abundance of the phylum Bacteroidetes and decreased the abundance of the phylum Bacteroidetes in rats (Yang M. et al., 2021). In addition, bile acids can also inhibit bacterial growth by increasing cell membrane permeability, inducing oxidative damage in bacterial DNA and altering protein conformations (Begley et al., 2005).

The metabolism of bile acids plays an important role in human metabolism, and in IBD, bile acids exhibit significant influence on the structure and composition of the intestinal flora. Their anti-inflammatory, intestinal barrier-enhancing, and immunomodulatory effects portend a possible future therapeutic modality for IBD, but research on their complex mechanisms and their use in the treatment of IBD and other diseases requires additional study. LCA, in particular, has been found to play an important role in suppressing inflammation and promoting intestinal stem cell proliferation and may be potentially therapeutic.

### 3.3. Tryptophan

Tryptophan, an essential amino acid, is an aromatic amino acid that cannot be synthesized by the human body and is an important source of tryptophan from food and some intestinal bacteria, as well as a precursor of 5-HT, melatonin, niacinamide, and vitamin B3 in the body (Agus et al., 2018; Lavelle and Sokol, 2020). Trp affects the body primarily through the KYN metabolic pathway, and tryptophan is a precursor for KYN, KNA/Kna/KA and other metabolites via IDO1 in intestinal cells (Salminen et al., 2021). Tryptophan can be metabolized to 5-HT and melatonin derivatives via TPH1 (Lavelle and Sokol, 2020; Salminen et al., 2021). Tryptophan can also be used to produce indole, IAAId, IAA, and other indole derivatives via intestinal flora (Lavelle and Sokol, 2020; Li X. L. et al., 2021). In addition, intestinal flora can also produce indole derivatives such as indole, IAAId, and IAA from tryptophan to regulate various processes such as immunity, cell growth, reproduction, and secretion (Lavelle and Sokol, 2020).

In an analysis of stool samples from patients with IBD, tryptophan and IAA levels were found to be lower than normal, whereas Kyn levels were elevated (Lamas et al., 2016). In a continuous 3-year follow-up of UC and CD patients in Germany, serum tryptophan levels were significantly lower in IBD patients than healthy controls and were lower in CD compared with the UC group. Colon biopsies in the IBD group revealed significantly increased IDO1 levels, indicating increased metabolism of tryptophan to the KYN pathway and increases in the metabolite Quinolinic acid (Nikolaus et al., 2017). The indole derivative IPA also exhibited a decrease in IBD serum (Alexeev et al., 2018). Furthermore, a 2018 study confirmed that increased KYN was significantly associated with the degree of endoscopic inflammation in UC patients (Sofia et al., 2018). As early as the 1960s, researchers observed changes in 5-HT in the intestine of patients with IBD, notably decreased 5-HT in both UC and CD patients compared with normal subjects (Coates et al., 2017). However, in a 2015 report, 5-HT levels were higher in UC patients than in normal subjects (Sikander et al., 2015).

Tryptophan metabolites promote intestinal barrier repair. In one experiment, induction of the human enterocyte line HCT-8 with physiological amounts of indole was found to increase the expression of the gap junction proteins GJE1, GJB3, GJB4, GJA8, and muc1, enhancing the epithelial and mucus barriers while inhibiting the activation and secretion of IL-8 and NF-κB (Bansal et al., 2010). IPA alleviates DSS-induced colitis by increasing tight junctions and the muc2-influenced mucus barrier via Pregnane X Receptor (PXR), thereby promoting intestinal barrier integrity while inhibiting TNF-α secretion (Mani and Li, 2015; Lavelle and Sokol, 2020). A decrease in AhR ligands was observed in the gut microbiota of IBD individuals. IPA promoted the transcription of AhR, which binds ligands such as KYN and promotes the expression of IL-10, IL-22, IL-17, and IFN-γ, leading to the proliferation of T-regs and suppressing intestinal inflammation (Monteleone et al., 2011; Alexeev et al., 2018; Sun et al., 2020). KYNA, an important metabolite of KYN, binds to AHR in intestinal epithelial cells to stimulate IL-6 production, whereas in macrophages, IL-6 production is inhibited through the mediation of AHR (Wang D. et al., 2021). In addition, metabolites such as KYNNA and 5-HT can also inhibit inflammatory factors such as IL-1, IL-4, IL-6, and TNF-α through signal transduction and together maintain intestinal homeostasis (Elizei et al., 2017; Badawy, 2018; Gao et al., 2018; Szabo et al., 2018). 5-HT can also affect macrophage polarization through the upregulation of M2-related genes and the downregulation of M1 gene expression (De Las Casas-Engel et al., 2013). Xanthurenic acid (XANA) and kynurenic acid (KYNA) promote oxidative phosphorylation of intestinal epithelial cells, increase their proliferation, and mediate tissue repair directly by AhR and indirectly by the AhR-IL-22 axis. XANA and KYNA and promote mitochondrial metabolism of IECs as well as glycolysis of T cells, increasing T-cell activation and polarization to Th17 (Michaudel et al., 2022). *Pseudomonas roxellana* can increase the number of cupped cells and glycosylation of the mucin rockweed, produce the tryptophan metabolite indoleacrylic acid (IA), promote the function of the intestinal barrier and reduce inflammation (Wlodarska et al., 2017). *Lactobacillus bulgaricus* OLL1181 inhibits DSS-induced colitis by activating AhR (Takamura et al., 2011). High levels of 5-HT promote the production of inflammatory factors and tight junctions and the breakage of tight junctions by activating NOX2, making it easier for *E. coli* to invade (Banskota et al., 2017). In addition, IPA reversed the increased ratio of thick-walled bacteria/mimics in DSS mice, decreased the abundance of Aspergillus and increased the abundance of Lactococcus (Fu et al., 2022).

All the above studies suggest that tryptophan regulates inflammation, immunity, and intestinal homeostasis in the body in a variety of ways. There is a wide range of tryptophan derivatives, and more research is needed to further identify the mechanisms of action by which they work as well as to explore their role in IBD treatment.

### 3.4. Others

In addition to the above three classical metabolites, other metabolic molecules of *G. intestinalis* affect the intestinal environment. In UC patients, L-Arg is decreased, and L-Cit is increased in colonic tissue (Coburn et al., 2016). In patients with CD, leucine, lysine, valine, arginine, glutamine, and serine are decreased, and citrulline is increased (Scoville et al., 2018; Krzystek-Korpacka et al., 2020). This change suggests that some amino acids play a role in the pathogenesis of IBD. Putrescine production by enterobacteria increased the abundance of anti-inflammatory macrophages in the colon, and the polyamines produced improved the symptoms of DSS-induced colitis in mice (Nakamura et al., 2021). Five genera of fungi (Cyanobacteria, Clostridium, Enterococcus, Ruminococcus, and Tizards) produced large amounts of aromatic amines via aromatic amino acid decarboxylase (AADC) and promoted the production of 5-HT (Sugiyama et al., 2022).

Changes in metabolites, as bridge substances for enterobacterial interactions, are direct manifestations of the metabolism of the intestinal flora in the host, and the flora acts on the organism through metabolites. Future research lies in two directions: first, to explore the relationship between flora and metabolites, and second, to explore the relationship between metabolome and organism. Clarifying the molecular mechanism underlying flora metabolism is important for fecal transplantation and precise strain transplantation, among others. Exploring the molecular mechanism underlying the role of the metabolome and how to intervene in pathophysiological processes may identify therapeutic approaches such as probiotics, synbiotics, and postbiotics. In addition, metabolites can be used as markers of disease onset and play a diagnostic role. Relatives of IBD patients may also exhibit similar changes in metabolic profiles, and metabolites can help in the identification of high-risk groups and predict of disease course (Lavelle and Sokol, 2020).

## 4. IBD therapies based on microbes and metabolites

**Figure 1:**
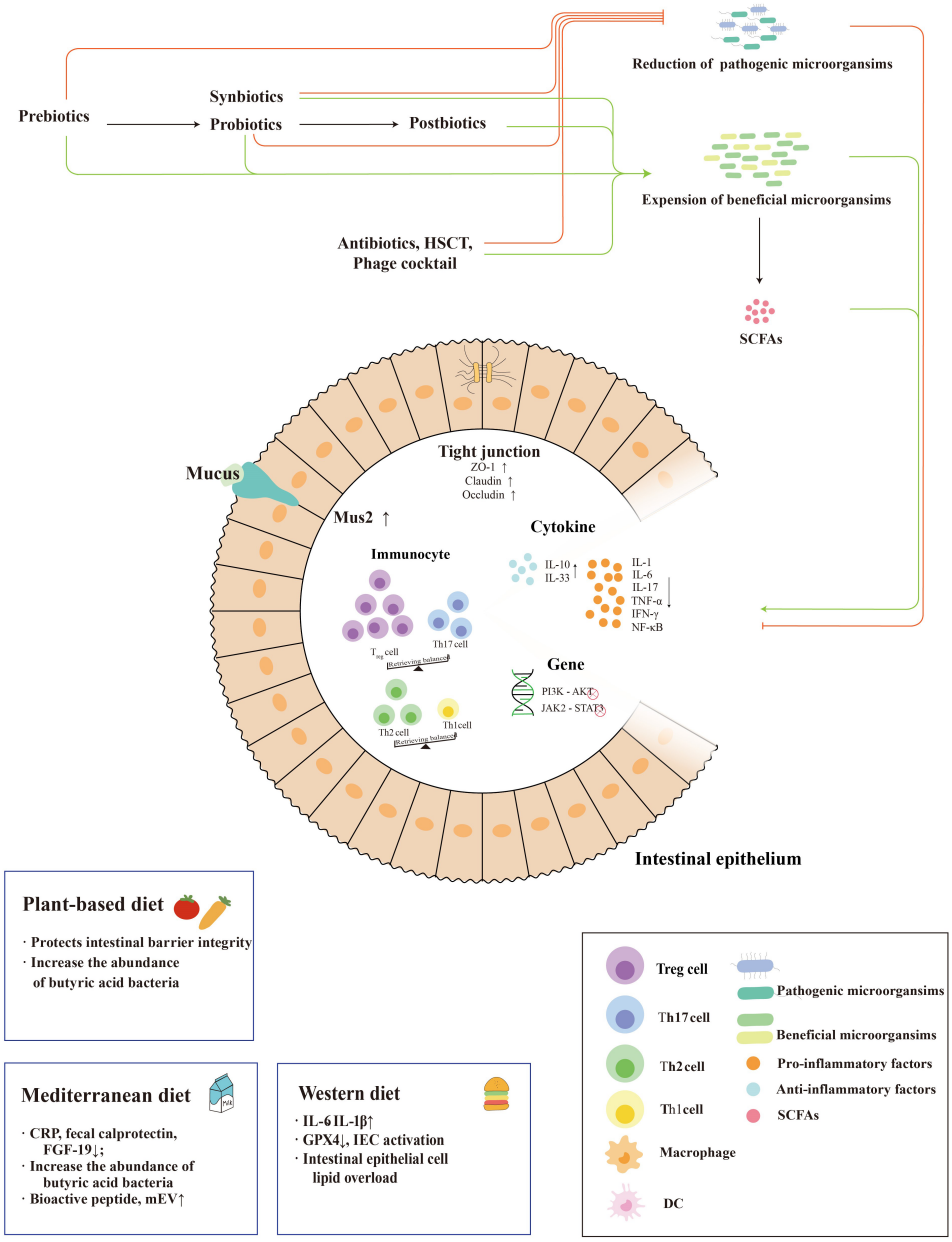
Mechanisms of microbiome-based therapies in IBD. The treatment modalities for IBD based on enterobacterial interactions are mainly shown in the figure. Systematic external intervention including probiotics, prebiotics, synbiotics and postbiotics regulate the intestinal barrier, intestinal anti-inflammatory and pro-inflammatory factor balance and immune cells to reduce the inflammatory response of IBD and promote its remission. In addition, they regulate the dominant strains of intestinal bacteria to reduce abundance of pathogenic bacteria and increase the abundance of beneficial flora. And three dietary patterns play different roles in IBD, as an anti-inflammatory factor or an pro-inflammatory factor.

IBD is closely related to the disturbance of intestinal bacteria, suggesting an underlying mechanism. Probiotics, prebiotics, and key metabolites with reduced inflammatory responses have become strategies to restore the balance of intestinal flora and reverse IBD. The following is a brief description of the currently proposed gut bacteria-based treatments for IBD (Figure 1).

### 4.1. Probiotics

Probiotics are live microorganisms that are supplemented from outside the body and are beneficial to the human body, balancing and stabilizing the intestinal microbiota, preventing intestinal infections, and stimulating the immune response (Auclair et al., 2015; Tamaki et al., 2016; Meng et al., 2021). Oral probiotics can regulate the intestinal flora in the body, maintain intestinal barrier function and regulate the host immune system and are widely used and promoted in food products (Champagne et al., 2018; Terpou et al., 2019; Jakubczyk et al., 2020). Several strains of bacteria are thought to potentially improve intestinal inflammation, including Bifidobacterium, Lactobacillus, and Mycobacterium.

*Lactobacillus plantarum* is resistant to acids and salts, is widely found in foods such as kimchi and is widely discussed in the treatment of IBD (Kawahara et al., 2015). DSS mice fed *Lactobacillus plantarum* exhibited an increase in the length of the colon and an increase in the actinomycetes content. Bacteria such as *Lactobacillus royale* that have been shown to be beneficial for IBD remission are increased compared with both healthy controls and DSS mice, suggesting that their effects on intestinal flora may be achieved by altering intestinal microecology (Yokota et al., 2018; Ding et al., 2021). A study by Pan et al. gave the same opinion, noting that *Lactobacillus plantarum* ZS62 increased colon length and inhibited colon atrophy in mice with IBD. The authors also observed mouse colon specimens and found that *Lactobacillus plantarum* improved DSS-induced cup cell destruction (Yokota et al., 2018). The levels of TNF-α, IFN-γ, IL-1β, IL-12, IL-8, and IL-6 in the blood of mice treated with *Lactobacillus plantarum* induced by DSS were lower than those in DSS mice, in contrast to untreated DSS mice, reflecting their decreased inflammation level (Zhang et al., 2018). IL-8 reduction was shown to have a protective effect on the colon of DSS mice; in human colonic epithelial cells, *Lactobacillus plantarum* had the effect of reducing TNF-α levels, and its downregulation of IL-8 was also demonstrated in the human colonic cell line HT-29 (Kim et al., 2012). However, at the cytokine level, the anti-inflammatory effect of IL-8 is weaker than that of salazosulfapyridine (Pan et al., 2021). Extracellular vehicles (EVs) produced by *Lactobacillus plantarum* produced a similar alleviating and inflammation-reducing effect, accompanied by a decrease in pro-inflammatory bacteria (Aspergillus) and an increase in anti-inflammatory bacteria (Bifidobacterium and Trichophytonaceae; Hao et al., 2021). Overall, the role of *Lactobacillus plantarum* in inflammation relief in the mouse colon is positive, but studies in humans remain limited to the colonic HT-29 cell line; more clinical studies are needed. Other similar strains, including *Lactobacillus rhamnosus*, have been shown to reduce TLR4 activation and attenuate NF-κB-mediated inflammatory responses through secreted proteins (Li Y. et al., 2020) but were not significantly different from the placebo group in clinical trials with respect to CD remission rates and magnitude of side effects (Limketkai et al., 2020). The clinical idea of probiotics as a therapeutic modality is not very consistent with its conception.

A probiotic combination of three bifidobacteria, four lactobacilli and *Streptococcus thermophilus* named Visbiome has been well studied and developed by pharmaceutical companies in recent years. Visbiome has been suggested as a monotherapy for children with mildly active UC who are intolerant of mesalazine or as an adjunctive therapy for children who have not achieved complete remission on standard therapy (Turner et al., 2012). Visbiome has been shown to modulate intestinal DCs, mediate microbial recognition and induce T-lymphocyte responses. No longer limited to studies in the laboratory, some clinical trials were promising; Visbiome reduced TLR-2 expression, increased IL-10 production, and reduced IL-12p40 levels in UC patients (Ng et al., 2010). A meta-analysis that included 22 randomized controlled trials pointed to a possible role for Visbiome in inducing remission in active UC, as demonstrated by a 56.2% rate of unobtained remission, which was statistically significantly different from placebo-treated patients (Ng et al., 2010). However, other randomized controlled trials with probiotics as clinical therapy included in the article also reported little evidence for the effectiveness of probiotics for the treatment of CD, either in inducing remission or preventing relapse, demonstrating the immaturity of probiotics as clinical therapy.

Probiotics come strictly from outside the gut; however, certain bacteria that have been shown to be present in the gut itself and have a protective effect on the gut have also teen supplemented as a form of IBD mitigation and may have better adaptive survival owing to their presence in normal organism (Wang G. et al., 2021). *Lactobacillus royi* is consistently considered to alleviate DSS-induced IBD, even though different studies have used different measures of stool consistency, blood in stool, and control of symptoms such as weight loss and colon length (Wang et al., 2020; Wang G. et al., 2021). Consistently, the levels of TNF-α, IL-1β, and IL-6 in mouse colonocytes were found to be decreased, and the same changes were observed in the HT-29 cell line (Wang et al., 2020; Wang G. et al., 2021). In experiments using *Lactobacillus royi* enemas in children with active UC, endoscopic Mayo scores were significantly lower, and IL-10 was significantly higher, whereas IL-1β, TNFα and IL-8 were significantly lower in the experimental group compared with the control group (Oliva et al., 2012).

In addition, engineering improved strains is a new idea for probiotic treatment of IBD. Benjamin et al. developed an engineered probiotic based on yeast expressing the human P2Y2 purinergic receptor, which activates purinergic signaling and promotes intestinal inflammation and pathology by increasing the sensitivity of eATP.

### 4.2. Prebiotics

Prebiotics are “a substrate that is selectively utilized by host microorganisms conferring a health benefit” (Gibson et al., 2017) that is mainly divided into polyols (sugar alcohols), oligosaccharides and soluble fiber derived from legumes, fruits, vegetables, milk, etc. (Mohanty et al., 2018). Prebiotics are derived from legumes, fruits, vegetables, and milk.

The most commonly used prebiotic treatments for humans are currently based on oligofructose-rich inulin (OF-in), fructo-oligosaccharides (FOS), and inulin. In a 2007 study, healthy volunteers taking OF-in had a significant increase in total fecal bifidobacteria (De Preter et al., 2007). In contrast, the administration of OF-in to CD patients increased the abundance of *Bifidobacterium longum* and significantly decreased *R. gnavus*; on the other hand, it also increased the fecal acetaldehyde and butyrate content (Joossens et al., 2012; De Preter et al., 2013). FOS increased the number of bifidobacteria and decreased the number of *Clostridium difficile* cluster XI and *Clostridium difficile* in the DSS-induced mouse model, in addition to improving the symptoms of colitis, inhibiting proinflammatory factors such as IL-6, promoting IL-10, enhancing the intestinal barrier, and improving the balance of Treg/Th17 cells. However, unlike animal studies, in a randomized double-blind trial of CD patients taking FOS, no changes were observed in fecal bifidobacteria, *Pseudomonas putida* or clinical symptoms, except for the observed decrease in IL-6 and increase in IL-10 (Benjamin et al., 2011; Koleva et al., 2012; Wang et al., 2022). In HLA-B27 rats fed inulin, a decrease in fecal *Clostridium difficile cluster* XI and *Clostridium difficile* was observed (Koleva et al., 2012). When inulin was administered to patients with UC, the patients exhibited showed a decrease in Mayo scores and fecal calprotectin, a reduction in gastrointestinal distention and an increase in butyrate and Bifidobacterium and Lactobacillus abundance (Valcheva et al., 2019). A recent study reported that inulin intake led to alterations in microbial metabolism, particularly increased CA expression, production of IL-33, and activation of ILC2s, leading to an increase in tissue eosinophils and promoting type 2 inflammation (Arifuzzaman et al., 2022).

In addition to the three most widely used prebiotics, LBPs *(Lycium barbarum polysaccharide)* and LbGP *(Lycium barbaru Glycopeptide)* from *Lycium barbarum* have high nutritional value, alleviating colonic inflammation, promoting SCFA production, promoting intestinal barrier repair, reducing ω-6 polyunsaturated fatty acids and amino acids in DSS mice, increasing Lactobacillus and decreasing the abundance of Lactobacillus, Escherichia, -Shigella, and Parabacillus (Huang et al., 2022; Sun et al., 2022). Sodium butyrate has a therapeutic effect on IBD, but it has a sour cheese-like fatty odor that makes it difficult for patients to take directly. Therefore, scientists obtained xylan butyrate ester (XylB) by esterifying linear xylan extracted from maize with butyrate, which increased butyrate content, decreased GPR109A expression in colon tissue, increased the number of CD4FoxP3Treg cells in mice, decreased CD4 IL-17ATh17 cells in the spleen, modulated the AMPK-mTOR signaling pathway and activated the autophagic pathway (Zha et al., 2020). In addition, extracts *of Rubia cordifolia* and *L. riboside*, as novel prebiotics, have been shown to alleviate the symptoms of colitis in DSS mice by inhibiting the IL-6/JAK2/STAT3 inflammatory pathway and NLRP3 inflammatory vesicles, restoring Th1/Th2 and Th17/Treg balance, among other mechanisms (Qin et al., 2022; Zhu et al., 2022). Aloin A also inhibits apoptosis and promotes the proliferation of colonic epithelial cells and barrier repair (Zhang R. et al., 2022).

In addition, Scutellaria baicalensis (Cui et al., 2021), mushroom (Kanwal et al., 2020), yam (Li P. et al., 2020), purple potato (Gou et al., 2019), konjac (Liu et al., 2016), green tea (oral; Wu et al., 2021), cranberry (Cai et al., 2019), orange pectin (Ishisono et al., 2019), quercetin (Lyu et al., 2022), Ganoderma lucidum polysaccharide (GLP; Xie et al., 2019), potato natural starch (PS), pea starch (PEAS), yam starch (CYS; Xu et al., 2021), tea flower polysaccharide (TFPS; Chen et al., 2020), FBTPS-3, and Fulbright tea purified polysaccharide (FBTPS-3; Chen et al., 2022) have been explored. The new prebiotics, such as Phytophthora capsulatum, have an important role in the treatment of colitis. Plant-processed products such as freeze-dried muscadine grapes (FMGs) or dealcoholized muscadine wine (DMW) may also play a similar role (Li R. Q. et al., 2020).

In recent years, scientists have made many research advances in the treatment of IBD with prebiotics, but they are almost superficial, which remains a challenge to clarifying further mechanism of action. Additionally, in clinical studies on patients, classical prebiotics such as inulin, FOS, and OF-in are still used, so more new prebiotics need to be verified urgently, and greater clinical and animal experiments are required to promote research moving from animal models to the clinic to bring real therapeutic effects.

### 4.3. Synbiotics

Synbiotics are “a mixture comprising live microorganisms and substrate (s) selectively utilized by host microorganisms that confers a health benefit to the host.” In studies of synbiotics, Lactobacillus, Bifidobacterium and Streptococcus spp. are often used as the probiotic fraction, with galacto-oligosaccharides, inulin or fructo-oligosaccharides selected as substrates (Swanson et al., 2020).

The effect of synbiotics on symptoms such as inflammatory infiltration and quality of life in the colon of patients with IBD, especially UC, is more pronounced than that of probiotics or prebiotics alone (Fujimori et al., 2009; Ishikawa et al., 2011; Zhang et al., 2021). This relationship was also illustrated in a 2020 study in which *Bifidobacterium infantis* and oligosaccharide xylooligosaccharide (XOS) reduced disease activity index (DAI), TNF-α, and IL-1β in DSS-induced mice treated with probiotics, prebiotics, and synbiotics, but IL-10 increased only with synbiotic treatment (Sheng et al., 2020). *Bifidobacterium longum* and Synergy 1 are the classical combinations of synbiotics. Furrie et al. (2005) used this combination for 1 month in UC patients and found that TNF-α and IL-1 α decreased significantly after 1 month, but IL-10 did not change significantly, human beta defensins2, 3 and 4 increased (Furrie et al., 2005). In 2010, Steed et al. also treated CD patients with this synbiotic for 6 months and observed no significant changes in IL-18, INF-γ or IL-1β in colonic tissue and a significant decrease in TNF-α expression at 3 months but not at 6 months (Steed et al., 2010). This finding indicates that *Bifidobacterium longum* and Synergy 1 synbiotics played an anti-inflammatory and flora-regulating role and have a better therapeutic effect on CD patients in the short term, which is much less than that on UC patients yet. Oligofructose and probiotic mixtures made into synergy preparations and used in patients with UC were found to exhibit reduced Truelove-Witts clinical activity index and colitis activity index (SCCAI) and were more effective in patients diagnosed more than 5 years ago (Amiriani et al., 2020). One study treated DSS mice with a variety of probiotic mixtures with prebiotics to form a synbiotic that alleviated clinical signs of colonic shortening and weight loss, increased MUC2 and tight junction (TJ) proteins, increased SCFA production, decreased TNF-α and IL-6, increased elevating IL-10, inhibited the Akt/p70S6K/mTOR inflammatory pathway, upregulated T-bet, and promoted the colonic innate immune response. Furthermore, fecal Lactobacillus, Akkermansia, Lacotbacilum, Bifidobacterium, and Ackermania were increased, and *Mucor spirochetes* and Bacteroidetes were decreased (Pistol et al., 2019; Son et al., 2019; Liu et al., 2020; Liao et al., 2021; Fei et al., 2022).

Long-term use of mesalazine can cause side effects such as nausea, vomiting, muscle pain, and interstitial nephritis. Therefore, some scientists have used mesalazine as a colloidal kernel with guar gum, xanthan gum and probiotics (*Lactobacillus acidophilus*, *Lactobacillus rhamnosus*, *Bifidobacterium longum* and *Saccharomyces boulardii*) to form a synergistic element that allows mesalazine to target the colon specifically. This approach avoids its side effects and allows mesalazine to act as an inhibitor of inflammation and to heal colonic ulcers in UC mice (Kaur et al., 2017). In addition to IBD, synbiotics also have significant therapeutic effects on colitis-related IBS and colon cancer, such as improving clinical symptoms, enhancing the intestinal barrier, inhibiting pro-inflammatory factors, promoting anti-inflammatory factors and regulating intestinal bacterial balance (Dos Santos Cruz et al., 2020).

Currently, synbiotic formulations have been extensively studied both in animal models and at the human level, demonstrating better therapeutic effects than their components. However, the mechanism remains unclear. We found that the therapeutic effects of synbiotics were different in UC and CD patients and different periods of the disease. Therefore, in further studies, more attention should be given to the mechanism of action of synbiotics in UC and CD as well as to the subtype of synbiotic components. In addition, the therapeutic effects of active, remission, short-term, and long-term treatment need to be further investigated. Treatment plans should be formulated by different combinations of synbiotics in different periods, and individualized treatment with different methods for different patients should be adopted.

### 4.4. Postbiotics

Postbiotics are the “preparation of inanimate microorganisms and/or their components that confers a health benefit on the host” (Salminen et al., 2021). Postbiotics primarily include the metabolites of probiotics in cell-free supernatants, such as SCFAs, vitamins, organic acids, neurotransmitters, amino acids, and terpene derivatives, which can be obtained from probiotics and used directly in the organism to regulate the immunity and metabolism (Nataraj et al., 2020).

SCFAs are an important class of postnatal elements with therapeutic effects in patients with IBD, but their therapeutic effects have also been reported to be limited to UC and less useful for CD treatment (Peng et al., 2022). We have already discussed the mechanisms by which SCFAs are associated with IBD, and several studies have demonstrated that SCFAs, especially butyrate, exhibit therapeutic effects on IBD. By identifying differential gene expression in pediatric and adult patients with UC, butyrate was shown to be associated with UC pathogenesis and is expected to be further investigated as a therapeutic tool (Zhou et al., 2021). Luceri et al. found that 30-day sodium butyrate enemas alone improved endoscopic scores, reduced intestinal mucosal atrophy, and promoted mucosal repair in patients with IBD (Luceri et al., 2016). A recent prospective, randomized, placebo-controlled multicenter study lasting 12 months reported that administration of sodium butyrate to patients aged 6 to 18 years with CD or UC lead to remission rates of 62% for CD and 76% for UC, with no significant difference in remission between the two groups (Pietrzak et al., 2022). In addition, butyrate alleviated intestinal inflammation, decreased iNOS, CCL2, TNF and IL-6 production, strengthened the intestinal barrier, alleviated colonic shortening, and reduced DAI scores, resulting in increased intestinal bacterial diversity, decreased abundance of *Bacillus mimicus* and *Bacillus deformans*, and increased Firmicutes in DSS-induced mice (Simeoli et al., 2017; Dou et al., 2020). Microencapsulated sodium butyrate (BLM) supplementation of the traditionally used drug mesalazine was administered to UC patients, and Mayo Partial Score (MPS) ≤ 2, Short Inflammatory Bowel Disease Questionnaire (SIBDQ) and visual analog scale (VAS) scores were used as the basis for treatment success. After 12 months of treatment follow-up, 83.3% of patients using BLM supplementation achieved MPS ≤ 2, compared with 64.1% of patients using mesalazine only. SIBDQ and VAS scores improved in 72.2% of patients using BLM supplementation compared with 23.8% of the control group (Vernero et al., 2020).

Cell-free supernatants of *Lactobacillus acidophilus*, *Lactobacillus casei*, *Lactococcus lactis*, *Lactobacillus royi* and *Saccharomyces boulardii* contain multiple postbiotic elements that downregulate PGE-2 and IL-8 in human colonic epithelial HT-29 cells and exert anti-inflammatory effects in isolated venous blood human macrophages by downregulating TNF-α and upregulating IL-10 (De Marco et al., 2018). Feeding lambs the cell-free supernatant of *Lactobacillus plantarum* RG14 increased the length and width of the rumen of lambs and increased the expression of the tight junction proteins, Tight junction protein 1(TJP-1), Claudin 1(CLDN-1) and Claudin 4(CLDN-4), which strengthened the intestinal barrier. Treatment also downregulated the proinflammatory factors IL-1β, IL-10, and TNF (Izuddin et al., 2019). Protein HM0539, extracted from *Lactobacillus rhamnosus* GG supernatant, improved DSS-induced colitis in mice and increased body weight and colon length compared to the no-treatment group. Additionally, the levels of zonula occludens-1 (ZO-1) and intestinal mucin (MUC2) increased, leading to decreased intestinal permeability (Gao et al., 2019).

The human body cannot produce vitamins and must rely on food or enterobacteria. Enterobacteria can produce microbial K, microbial B12, and many other microorganisms for human use. In DSS mice fed VB12, it alleviated colonic shortening and damage to the intestinal epithelium and altered the intestinal microbial ecology (Lurz et al., 2020). Conjugated linoleic acid (CLA) reduced DAI in DSS-induced mice, improved inflammatory infiltration of the mucosa, and decreased the expression of TNF-α and MCP-1 (Bassaganya-Riera et al., 2012). Gut flora can also influence gut and brain function by producing various neurotransmitters such as GABA, HT, catecholamines, dopamine and acetylcholine (Bassaganya-Riera et al., 2012).

At present, postbiotics are primarily studied in clinical practice in SCFAs, similar to prebiotics and postbiotics, and more research is needed to elucidate their mechanisms of action. In addition, whether postbiotics have side effects and how they maintain stability needs to be explored.

### 4.5. Dietary therapeutics

Dietary composition depends on culture and many other factors, taking indispensable effect on interaction between intestinal flora and IBD. Common dietary structures include the Western diet, plant-based diet, and Mediterranean diet.

One cross-sectional analysis demonstrated that Crohn’s disease patients preforms low Mediterranean dietary preferences. Only 1.1% of men and 1.8% of women meeting the criteria for a Mediterranean diet in patients with Crohn’s disease, exhibiting reduced intake of monounsaturated fat (MUFA), omega-3 polyunsaturated fat (PUFA), magnesium, phosphorus, zinc, potassium, and vitamins C, D, thiamin and niacin (Taylor et al., 2018). In a prospective study of 1,042 patients, a 6-month Mediterranean diet intervention reduced BMI, waist circumference, serum LDL, CRP, fecal calprotectin levels, and IBD activity in IBD patients (Chicco et al., 2021). A clinical research containing dietary habits of 454 IBD patients provided one possible mechanism that the Mediterranean diet was negatively associated with FGF-19, and that the farnesol X receptor-FGF-19 axis is considered a potential therapeutic target for IBD. Activation of this axis is commonly thought to inhibit intestinal inflammation and protect the intestinal barrier in IBD patients (Gadaleta et al., 2011; Chicco et al., 2021). As one proportion of the Mediterranean diet, milk is a source of many bioactive peptides, including MBCP (De Simone et al., 2011), which has been shown to promote the formation of tight junction repair in Caco-2 cells, reduce intestinal permeability, and attenuate the pro-inflammatory effects of TNF-α by modulating the NF-κB pathway (Tenore et al., 2019). Milk extracellular vesicles (mEVs) alleviate DSS-induced colitis by reducing TNF-α and IL-6 expression (Reif et al., 2020; Zonneveld et al., 2021).

The Western diet has been discussed extensively in studies of recipes for patients with IBD, which is thought to promote intestinal inflammation in IBD patients. A high-protein diet at the same caloric level resulted in higher IBD activity and greater intestinal mucosal damage in mice, as well as elevated levels of IL-6 and IL-1β, among others, whereas a moderate-protein diet with upregulated Gpx2 gene expression in mice enhanced tissue repair, as evidenced by a decrease in Caspase-3-expressing epithelial cells; this finding is thought to be due to its induction of butyrate-producing resulting from bacterial colonization (Vidal-Lletjós et al., 2019). Reduced intake of processed meat and refined carbohydrates and diets with higher water content are associated with reduced levels of IBD activity (Limketkai et al., 2022), and will not lead to a decrease in muscle mass or strength according to one clinical research on pediatric Crohn disease patients (Lee et al., 2018). Administration of a high-fat diet to mice accelerated weight loss in mice with DSS-induced colitis, suggesting that a high-fat diet may exacerbate IBD activity (van der Logt et al., 2013; Lee et al., 2020). One possible mechanism is that ω-6 polyunsaturated fatty acids (PUFAs) activate cytokine responses to IECs restricted by GPX4, leading to lipid peroxidation and intracellular iron deposition and triggering focal granuloma-like neutrophilic enteritis (Mayr et al., 2020). Notably, promoting the release of lipids from intestinal cells with Cideb, a lipid droplet-associated protein, attenuated triglyceride accumulation and oxidative stress in Caco-2 cells and instead attenuated the intestinal inflammatory response, suggesting that the effect of a high-fat diet on the degree of activity in IBD may be achieved through lipid overload in intestinal epithelial cells (Sun et al., 2017).

Plant-based diets (PBD), which include vegan and lacto-ovo-vegetarian diets, are characterized by lower protein, higher fiber and polyunsaturated fatty acids (PUFAs) intake within the recommended range and have been shown to play a role in the prevention and treatment of metabolic diseases such as type 2 diabetes (Chen Z. et al., 2018; Baden et al., 2019; Molina-Montes et al., 2020; Craig et al., 2021; Neufingerl and Eilander, 2021). Increased intake of fruits and vegetables can reduce the activity level of IBD, reduce symptoms during the active phase of IBD patients and maintain the integrity of the intestinal barrier, suggesting that a plant-based diet may have a more systemic role (Limketkai et al., 2022). Increased abundance of butyrate-producing bacteria was found in the intestine of plant-based dieters, which may be one mechanism underlying their protective effect on the intestinal mucosa (Singh et al., 2017; see later, Prebiotics and IBD for details). In several cases of complex IBD, the activity of IBD was significantly reduced by the administration of a lacto-ovo-vegetarian, or vegan, diet (Chiba et al., 2019; Sandefur et al., 2019). However, there is controversy as to whether full PBD should be adopted in patients with IBD, and there may be problems with patient cooperation; both of the aforementioned clinical reports used this diet only during specific periods of disease, and more clinical trials are needed to validate it (Chiba et al., 2019; Sandefur et al., 2019).

Low FODMAP diet (LFD) is the recommended diet for patients with IBS (Staudacher and Whelan, 2017). The randomized controlled clinical trial by Cox et al. reported that patients on the LFD diet exhibited higher health-related quality of life scores than those on the control diet (Cox et al., 2020). Another study also reported improvements in overall abdominal symptoms in CD and UC patients on the LFD diet (Gearry et al., 2009). However, the long-term implementation of a low FODMAP diet can itself produce health problems because fruits such as apples, pears, and drupes are rich in fructose and other FODMAPs. Some foods with intestinal mucosal protective properties, such as beans and soy products, are also high in FODMAP, as are several vegetables, including onions and garlic (Hills et al., 2019; Basson et al., 2021).

Various nutrients are present in different dietary structures, but some of the nutrient components should be discussed separately. Vitamin D is thought to correlate with the inflammatory response and has a positive correlation with TNF-α, fecal calprotectin, and CRP, reflecting the degree of disease activity in IBD (López-Muñoz et al., 2019). The prevalence of IBD with comorbid osteopenia and osteoporosis is 37%, which may be associated with reduced vitamin D levels (Bryant et al., 2018). Retinoic acid (RA), one of the active products of Vitamin A, exhibits an anti-inflammatory phenotype by promoting CD4^+^T-cell differentiation and production of TGF-β and IL-10 and by inhibiting the differentiation of both Th1 and Th17 cells, and alleviates DSS-induced IBD (Coombes and Powrie, 2008). However, in the mucosa of UC patients, RA levels were positively correlated with IL-17 and IFNγ expression on CD4 and CD8 T cells and negatively correlated with IL-10-expressing CD4^+^ T cells, suggesting dual effect of RA in colon inflammation (Rampal et al., 2021). A study reported that 19.6% of CD and 21.6% of UC were found to exhibit iron deficiency anemia associated with malabsorption in patients with IBD (Madanchi et al., 2018). However, it is dramatic that iron supplementation is positively correlated with the activity of IBD. An iron sulfate-free diet prevented the development of chronic ileitis in a mouse model of DSS-induced Crohn’s disease, compared with systemic iron supplementation (Werner et al., 2011). The authors suggest that intraluminal iron may directly affect IEC function or produce a pathological environment in the gut that triggers stress-related apoptosis of epithelial cells through changes in microbial homeostasis (Werner et al., 2011). Overall, the effect of each component on disease progression in IBD is relatively well established, but the specific recommended dose of each component remains unclear and requires further experimental demonstration.

### 4.6. Others

In recent years, new therapeutic approaches based on gut microbes and their metabolites have been discovered for the treatment of IBD.

Antibiotics are a class of traditional drugs that alter the macroecology of intestinal bacteria by inhibiting the growth of intestinal bacteria to improve IBD (Buttimer et al., 2022). Antibiotics such as ciprofloxacin, metronidazole, clarithromycin, and rifaximin can improve and relieve CD symptoms, such as perianal fistula in CD, but their effects are not particularly pronounced. Whether antibiotic use is beneficial for the maintenance therapy in CD is uncertain, and more research is needed (Burke et al., 1990; Townsend et al., 2019). Oral tobramycin and metronidazole have significant therapeutic effects in patients with UC, but metronidazole and ciprofloxacin do not affect UC remission (Burke et al., 1990). Vancomycin and ampicillin alter intestinal bacterial diversity and intestinal bacterial composition (Ubeda et al., 2010). Oral vancomycin and gentamicin are effective in 80% of children with IBD (Lev-Tzion et al., 2017). However, antibiotics can cause gastrointestinal disorders, tendonitis, thrush, drug resistance, neuropathy and many other side effects (Nitzan et al., 2016).

miRNA regulates NF-κB, STAT3, PI3K/AKT and other pathways to modulate the synthesis and release of inflammatory factors and promote the repair of the intestinal mucosal barrier and epithelial barrier, thereby inhibiting colitis in mouse models and alleviating intestinal inflammation in IBD patients in the clinical setting (Innocenti et al., 2022).

M13/nLNP, a lipid nanoparticle-encapsulated drug, alleviated clinical signs of IBD, reduced intestinal mucosal ulcers, and decreased TNF-α and IL-6 mRNA levels in experiments on IL-10KO mice. *In vitro* fermentation of mouse feces revealed anti-inflammatory effects with increasing concentrations of fecal lactobacilli and decreasing bacilli populations (Yang et al., 2022). IL-22 mRNA was encapsulated in a novel nanoparticle and fed to a mouse model of acute colitis, and recovery of colonic length and tissue damage was observed, with concomitant reductions in TNF-α, IL-6 and IL-1β expression (Sung et al., 2022).

In recent years, phage cocktail therapy has been widely studied in IBD. Using a mixture of six phages, researchers have reduced the number of *Escherichia coli* and *Enterococcus faecalis* (important members of IBD pathogenesis) by acting as growth inhibitors *in vitro*, but this effect was not observed in mice, suggesting that the translation from *in vitro* to *in vivo* requires considerable study (Buttimer et al., 2022).

Significant remission was observed following hematopoietic stem cell transplantation (HSCT) in patients with severe CD who were not treated with antitumor necrosis factor, with decreasing effects over a 5-year time span (Burt et al., 2010). HSCT relieved clinical symptoms and improved endoscopic scores in patients with CD but also led to complications such as infection (Zhang H.-M. et al., 2022; Zhang L. et al., 2022). Intravenous administration of adipose stem cells (ADSCs) in DSS mice also resulted in relief of clinical signs, reduction of inflammation levels and restoration of intestinal flora balance (Zhang R. et al., 2022). In addition, Mesenchymal stem/stromal cells (MSCs) can also alleviate symptoms of colitis, improve lymphatic drainage, inhibit macrophage infiltration, and improve IBD (Wang et al., 2016; Zhang H.-M. et al., 2022; Zhang L. et al., 2022).

## 5. Conclusion and perspectives

Researchers have conducted extensive studies on the pathogenesis of gut microbes in IBD and found that they play an important role in the inflammatory response and help regulate immunity, gene expression, and cellular proliferation. Therefore, therapeutic tools targeting gut microbes have become an emerging research direction for IBD. In this paper, we describe the mechanisms and therapeutic strategies underlying the use of intestinal flora and their metabolites in IBD from the perspective of enterobacterial interactions.

Probiotics, prebiotics, synbiotics, postbiotics, and dietary intake have demonstrated the regulatory effects of several substances related to intestinal bacteria on the gut. Their varied effects include the regulation of inflammatory factor secretion, reductions in intestinal and systemic inflammation, regulation of gene expression, strengthening the intestinal barrier, regulating Th and Treg cells and enhancing immunity, improving clinical symptoms, and regulating the homeostasis of the intestinal environment. In addition, antibiotics and FMT also have important therapeutic effects on IBD.

However, the current study of IBD therapeutics is limited, and there is a need for additional breakthroughs. In the treatment of IBD, anti-inflammatory drugs and immunosuppressants are commonly used at present, which have a wide range of effects and obvious side effects. Therefore, precise targeted therapy has become a promising direction. Synthetic nanoparticles and medicinal plants derived EVs carrying the effective active phytochemicals (as prebiotics) can deliver drug directly to colon, which play a role in improvement of intestinal flora and anti-inflammation (Li et al., 2023). Therefore, dietary supplements such as probiotics and prebiotics, combined with traditional medicines and targetedly delivered with EVs have research potential in the treatment of IBD in the future. In addition, different combinations of probiotics and prebiotics may have a synergistic amplification effect. The imbalance of intestinal microbes is not only related to IBD, but also induces colon cancer to a certain extent. Therefore, it is also a research direction to study the relationship between intestinal microbial balance and colon cancer as well as to find microbial therapies to prevent colon cancer. Dietary supplements may have different effects on patients from different age groups, different regions, special populations such as pregnant women, infants, the elderly, different disease subtypes (such as UC, CD) and different stages of disease development (active phase, remission phase). This may be due to the differences in the gut microbes of different populations. In the research process, full consideration should be given to the research in different animals and populations to achieve more precise intervention effects (Ni et al., 2023). Apart from bacteria, virus and fungi also play a vital role in IBD, which regulating and shaping microbial communities in the gut (Federici et al., 2022; Sinha et al., 2022). Therefore, it is a direction to find further relationship between virus and fungi and intestinal flora to expect for new therapies.

In conclusion, more mechanistic studies on IBD and intestinal microorganisms are expected, as well as more retrospective and prospective studies with larger sample *in vitro*, *in vivo*, in animal models, and in the clinic, to explore more accurate pathogenesis, discover more effective IBD treatments, and find effective means to prevent untreated diseases with simple, effective, and accessible treatments to prevent and treat the disease.

## Author contributions

YW and GR: conceptualization. SL and KX: methodology. LC, AY, and ZX: investigation. SL and KX: data curation. YW, GR, SL, and KX: writing—original draft preparation and writing—review and editing. YC, XZ, MC, YT, WM, ZT, and SZ: supervision. YW: funding acquisition. All authors contributed to the article and approved the submitted version.

## Funding

This research was funded by the National Natural Science Foundation of China, grant number NSFC 82070571, National Defense Technology 173 Program, grant number 2021-JCJQ-JJ-1083, and Clinical Technology Innovation and Cultivation Program of Army Medical University, grant number CX2019JS222.

## Conflict of interest

The authors declare that the research was conducted in the absence of any commercial or financial relationships that could be construed as a potential conflict of interest.

## Publisher’s note

All claims expressed in this article are solely those of the authors and do not necessarily represent those of their affiliated organizations, or those of the publisher, the editors and the reviewers. Any product that may be evaluated in this article, or claim that may be made by its manufacturer, is not guaranteed or endorsed by the publisher.
